# *Angelica acutiloba* Kitagawa flower induces A549 cell pyroptosis via the NF-κB/NLRP3 pathway for anti-lung cancer effects

**DOI:** 10.1186/s13008-023-00102-w

**Published:** 2023-11-01

**Authors:** Yonghu Chen, Fangying Zhu, Xianhua Che, Yanwei Li, Ning Li, Zhe Jiang, Xuezheng Li

**Affiliations:** 1 Yanbian University Hospital, Yanbian University, Yanji, 133002 People’s Republic of China; 2 Changchun Institute of Biological Products Co., Ltd, Changchun, 130012 People’s Republic of China; 3 School of Traditional Chinese Materia Medica, Shenyang Pharmaceutical University, Shenyang, 110016 People’s Republic of China

**Keywords:** *Angelica acutiloba* Kitagawa flower, NLRP3 inflammasome, Anti-lung cancer, Pyroptosis, A549 cells

## Abstract

*Angelica acutiloba* Kitagawa, a traditional medicinal herb of the *Umbelliferae* family, has been demonstrated to have anticancer activity. In this study, we investigated the anti-lung cancer effects of two compounds extracted from *A. acutiloba* flowers: kaempferol-3-*O*-*α*-L-(4″-*E*-p-coumaroyl)-rhamnoside (KAE) and platanoside (PLA). MTT, cell colony formation, and cell migration (scratch) assays revealed that both KAE (100 μM) and PLA (50 μM and 100 μM) inhibited the viability, proliferation, and migration of A549 cells. Dichlorodihydrofluorescein diacetate assays showed that KAE and PLA also induced the generation of reactive oxygen species in A549 cells. Morphologically, A549 cells swelled and grew larger under treatment with KAE and PLA, with the most significant changes at 100 μM PLA. Fluorescence staining and measurement of lactate dehydrogenase release showed that the cells underwent pyroptosis with concomitant upregulation of interleukin (IL)-1β and IL-18. Furthermore, both KAE and PLA induced upregulation of NF-κB, PARP, NLRP3, ASC, cleaved-caspase-1, and GSDMD expression in A549 cells. Subsequent investigations unveiled that these compounds interact with NLRP3, augment NLRP3’s binding affinity with ASC, and stimulate the assembly of the inflammasome, thereby inducing pyroptosis. In conclusion, KAE and PLA, two active components of *A. acutiloba* flower extract, had significant anti-lung cancer activities exerted through regulation of proteins related to the NLRP3 inflammasome pathway.

**Supplementary Information:**

The online version contains supplementary material available at 10.1186/s13008-023-00102-w.

## Introduction

According to survey studies [1, 2], in China the incidence of lung cancer ranks first among cancers, accounting for 24.6% of cases, and the mortality rate of lung cancer is as high as 29.71%. Globally, the incidence of lung cancer is 11.4%, second only to that of breast cancer; however, it is the leading cause of cancer death, at 18% [3]. Lung cancer is divided into two main types [4]: non-small cell lung cancer (NSCLC) and the relatively less prevalent small cell lung cancer (SCLC). The NSCLC disease type comprises 85% of lung cancer cases [5], and its causative factors are diverse, including smoking, occupational dust exposure, environmental dust pollution, history of lung disease, and genetic influence. The pathogenesis is complex and heterogeneous [6, 7]. The early manifestations of lung cancer are symptoms common to general respiratory diseases, and it is unusual to observe disease-specific symptoms [8]. Therefore, most patients with lung cancer are diagnosed in the middle and late stages of disease, [9] missing the optimal timing for treatment. Therefore, there is an urgent need to investigate the pathogenesis of lung cancer and develop better treatment options.

It has been shown that pyroptosis is a type of programmed cell death characterized by high inflammation and the release of proinflammatory signals in parallel [10]. Pyroptosis and apoptosis are both programmed cell death processes, but they differ in the way cells undergo death. Pyroptosis is primarily triggered by inflammasomes, the Caspase family, and the GSDM family of proteins, with Gasdermin D (GSDMD) being a key component. In pyroptosis, GSDMD is cleaved and transferred to the cell membrane, forming a pore that leads to cell swelling and the release of cell contents. In contrast, apoptosis is mainly associated with the Bcl2 protein family and Caspase family. During apoptosis, the cell’s plasma membrane undergoes blistering, chromatin condensation occurs, but the membrane remains intact, and the cell volume decreases. Research has revealed that treatment with substances like cisplatin or adriamycin can increase GSDMD-dependent pyroptosis in human renal tubular epithelial and mesothelioma cell lines through the activation of caspase-1. Targeting Src to reactivate pyroptosis has proven to be pivotal in overcoming chemoresistance in lung and pancreatic cancer models [11]. Additionally, Shao et al. [12] have discovered that inducing pyroptosis through GSDMB enhances anti-tumor immunity. Nanotuners can activate pyroptosis, inducing cancer cell death and thereby enhancing anti-tumor efficacy while minimizing systemic side effects [13]. An increasing body of research emphasizes the crucial role of pyroptosis in inhibiting the growth of lung cancer cells [14]. Current research shows that caspase-1 cleaves GSDMD, activating it and, on the other hand, facilitating the maturation of IL-1β and IL-18 [15, 16]. As a result, Caspase-1 actively participates in the process of pyroptosis [17]. The NLRP3 inflammasome is a complex of at least three proteins: the sensor (NLRP3), the adaptor (ASC), and the effector (pro-caspase-1) [18, 19]. It is activated in two ways. In the first pathway, the initiation signal causes entry of nuclear factor (NF)-κB into the nucleus, which leads to increased levels of NLRP3, pro-IL-1β and pro-IL-18. The newly synthesised NLRP3 is involved in the assembly process of the inflammasome, which leads to caspase-1 maturation [20–22]. In an alternative pathway that directly activates inflammasome assembly, it was shown that reactive oxygen species (ROS) can mediate the pore-forming activity of GSDMD by directly targeting the caspase-1 proteins involved in shearing. Both exogenous and endogenous ROS-induced stimuli cleave GSDMD, leading to cell membrane rupture and pyroptosis. Lactate dehydrogenase (LDH), an enzyme stabilised in the cytoplasm, is normally only found within the cell. When pyroptosis occurs, the plasma membrane is ruptured and LDH is rapidly released into the extracellular space, thus reflecting the degree of pyroptosis [23]. Additionally, poly ADP-ribose polymerase (PARP) has been widely studied as an apoptotic effector protein, but it also reportedly mediates the degradation of DNA and participates in the regulation of pyroptosis [24, 25]. Hence, research simultaneously inquired into the influence of active ingredients on its expression by using PARP as one of the targets of NLRP3 inflammasome pathway-related proteins.

*Angelica acutiloba* Kitagawa is of the same botanical family (*Umbelliferae*) and genus as *Angelica sinensis* Diels (ASD) [26, 27], and is often used as an alternative to ASD in Chinese herbal medicine [28]. Currently, the dried roots of *A. acutiloba* [29] are commonly used to treat irregular menstruation, dysmenorrhea, blood deficiency amenorrhea, abdominal pain, and constipation [30, 31]. Furthermore, the continuous research on natural drug components of traditional medicines, which are gradually being explored for new pharmacologic effects [32], has revealed that the roots of *A. acutiloba* can alleviate inflammatory bowel disease [33], while its leaves have been shown to be effective in reducing hypertension [34]. However, the pharmacological effects of *A. acutiloba* flowers have not yet been studied, especially the anticancer effects. To this end, with reference to the tumour-related studies of ASD [35, 36], we investigated the specific mechanism of action of *A. acutiloba* flowers extract on lung cancer in vitro. For the first time, this study elucidates the mechanism behind KAE and PLA inhibition of non-small cell lung cancer (NSCLC) in the *A. acutiloba* flowers. These findings provide a new scientific basis for the development of natural anti-lung cancer drugs.

## Results

### Structural elaboration of compounds

**Fig. 1:**
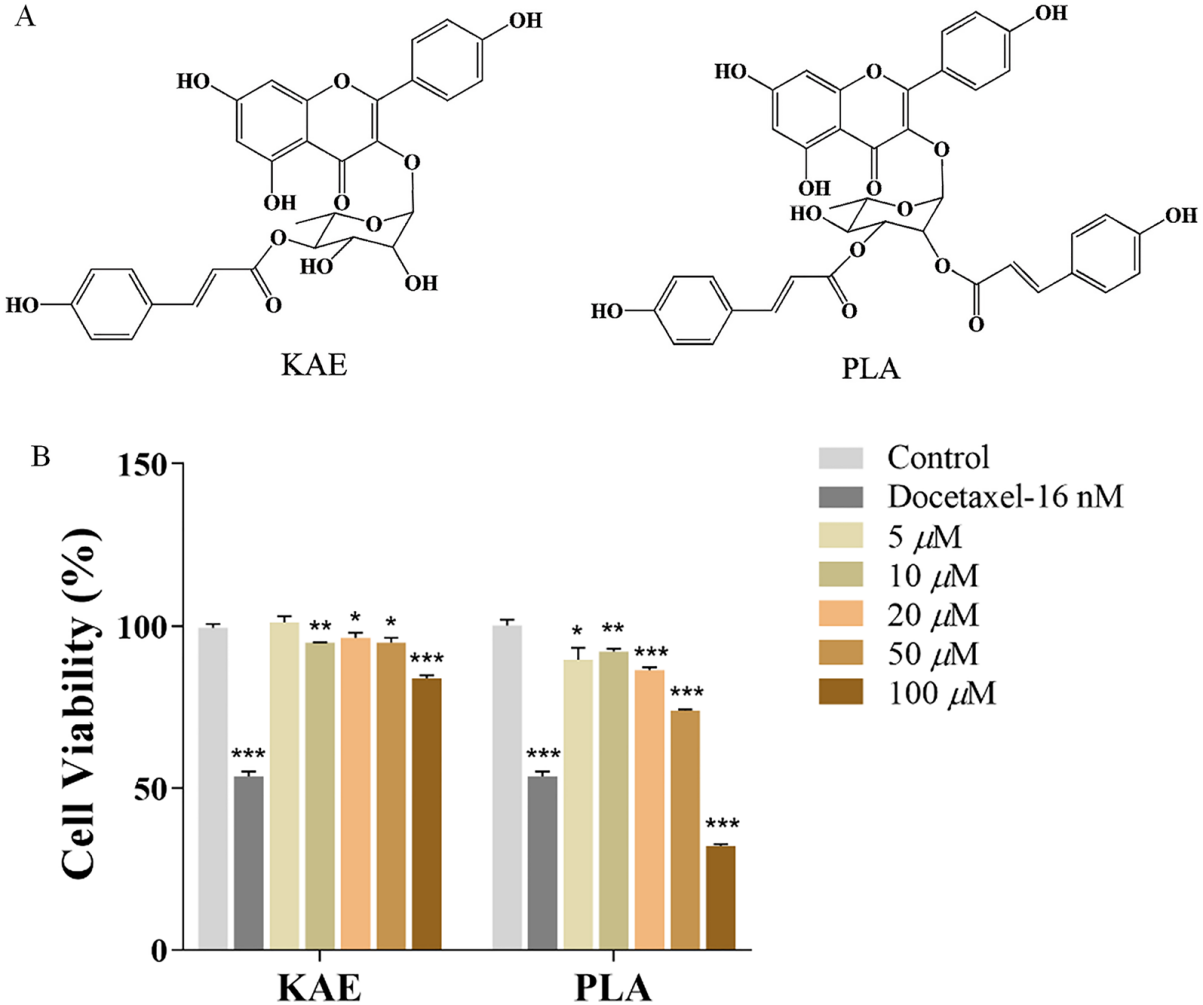
Two new *A. acutiloba* compounds with anticancer activity against A549 cells.** A** Chemical structures of KAE and PLA. **B** MTT assay of A549 cell viability under 24 h treatment with a negative control, docetaxel, or a range of concentrations of KAE or PLA; *P < 0.05, **P < 0.01, ***P < 0.001 vs. control group

The isolated compounds kaempferol-3-*O*-*α*-L-(4″-*E*-p-coumaroyl)-rhamnoside (KAE) and platanoside (PLA) were measured by NMR spectroscopy at ^1^H-NMR (300 MHz, CD_3_OD) and ^13^C-NMR (75 MHz, CD_3_OD), with chemical shifts expressed as *δ* (ppm) (Fig. 1A). These data were compared with the literature to determine that this was the first report of KAE and PLA isolation from the genus *Angelica* [37].

### KAE and PLA reduce A549 cell viability

First, we conducted a preliminary screening to determine whether KAE and PLA had anticancer activity against A549 cells. After testing a series of concentration gradients, we found that the anticancer activity of KAE and PLA was concentration dependent: 100 μM KAE, and 50 μM and 100 μM PLA, significantly inhibited A549 cell viability in vitro (Fig. 1B).

### KAE and PLA inhibit A549 cell proliferation

**Fig. 2:**
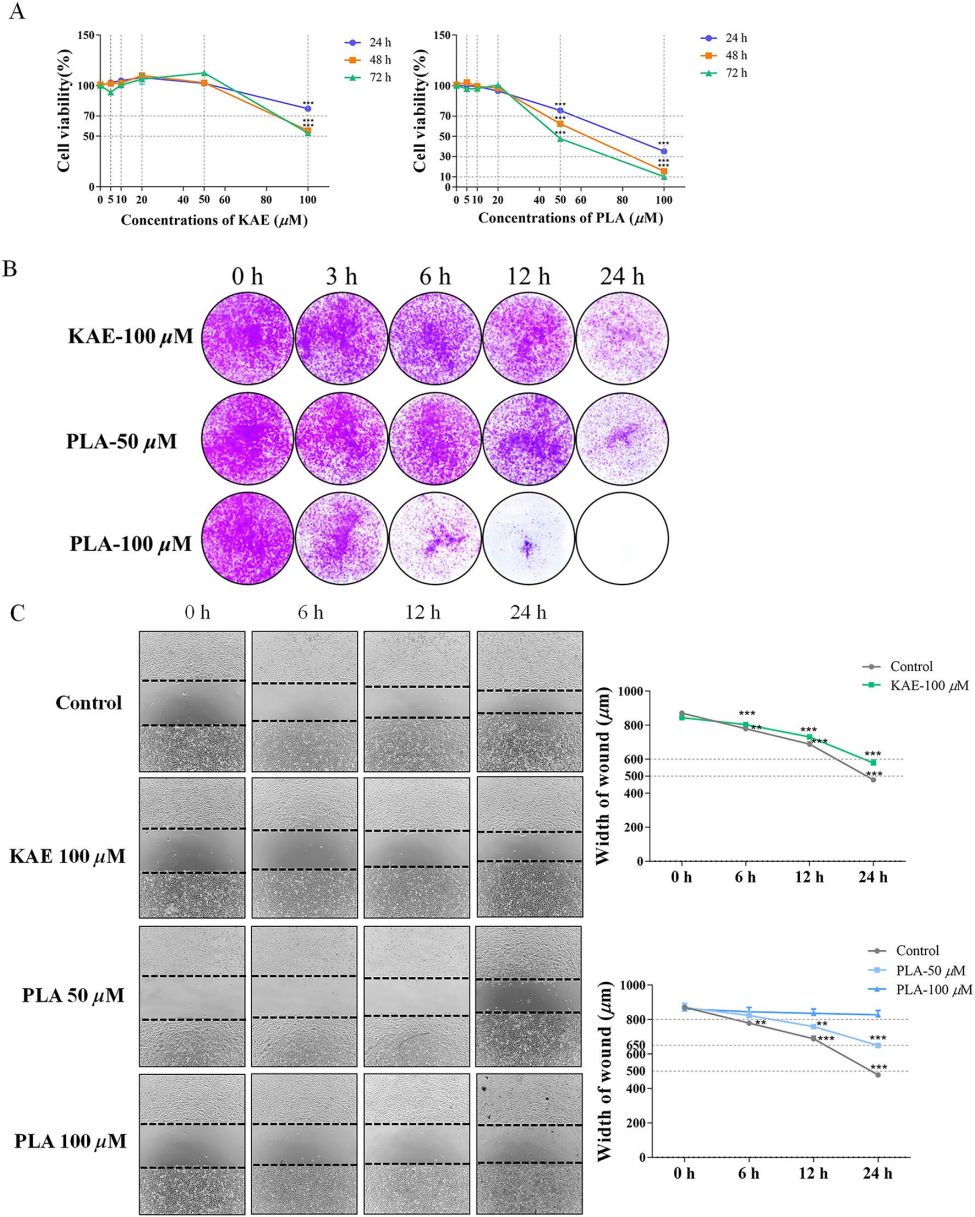
Inhibitory effects of KAE and PLA on proliferation and migration of A549 cells. **A** MTT assay of A549 cell viability showing the anti-proliferative effects of treatment with the indicated concentrations of KAE and PLA for 24, 48, and 72 h. **B** Colony formation assay of A549 cells under treatment with KAE (100 μM) or PLA (50 μM and 100 μM) for 3, 6, 12, and 24 h. **C** Cell migration (scratch) assay and quantitative analysis of A549 cells under treatment with 100 μM KAE or 50 μM and 100 μM PLA for 6, 12, and 24 h; **P < 0.01, ***P < 0.001 vs. control group

KAE and PLA also had anti-proliferative effects on A549 cells at high concentrations. KAE was most effective when administered at a concentration of 100 μM, while PLA showed strong anti-proliferative effects at both 50 μM and 100 μM; additionally, these effects were enhanced over time (Fig. 2A). Cell colony formation assays were also used to investigate the proliferation and colony-forming ability of A549 cells treated with KAE (100 μM) or PLA (50 μM and 100 μM). The resulting A549 cell colonies showed that both compounds had significant anti-proliferative effects and reduced colony formation in a time-dependent manner (Fig. 2B).

### KAE and PLA inhibit A549 cell migration

During the development of lung cancer, tumours show the ability to metastasise. Therefore, to evaluate the ability of KAE and PLA to inhibit the migration of A549 cells, we used a scratch assay to measure changes in the degree of healing at different time intervals. Both compounds inhibited cell migration. Compared with the control group, the scratch healing in the KAE- or PLA-treated A549 groups appeared to be slowed, with the degree of scratch healing increasing over time (Fig. 2C).

### KAE and PLA promote LDH release in A549 cells

**Fig. 3:**
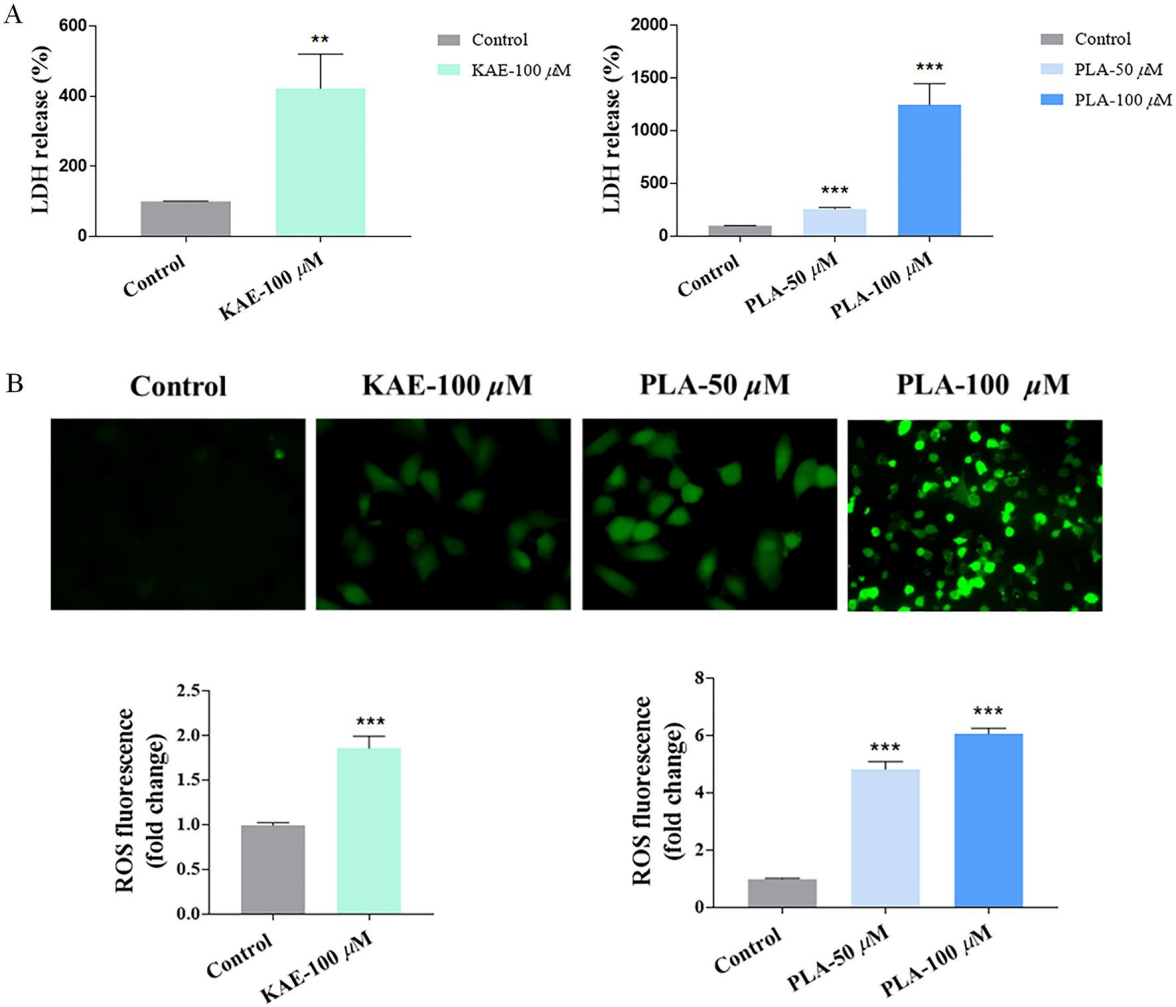
Promotion of the release of LDH and ROS by KAE and PLA in A549 cells.** A** and** B** LDH release assay (**A**) and DCFH-DA assay of ROS (**B**) in A549 cells treated with KAE (100 μM) or PLA (50 μM and 100 μM); **P < 0.01, ***P < 0.001 vs. control group

The activity of LDH in cell culture supernatants reflects the integrity of the cell membrane and may be used the estimate the degree of release of cell contents and thus the state of cellular health [38]. Therefore, we examined the effect of treatment with KAE or PLA on the level of LDH release into the cell culture medium (Fig. 3A). Compared with the untreated control, both compounds significantly promoted the release of LDH.

### KAE and PLA induce elevated ROS levels in A549 cells

Next, we examined the effect of treatment with KAE or PLA on the regulation of ROS production in A549 cells (Fig. 3B). Administration of KAE or PLA led to an increase in the number of cells with green fluorescence and enhanced the fluorescence intensity, indicating that both compounds increased the level of ROS release from A549 cells.

### KAE and PLA promote pyroptosis of A549 cells

**Fig. 4:**
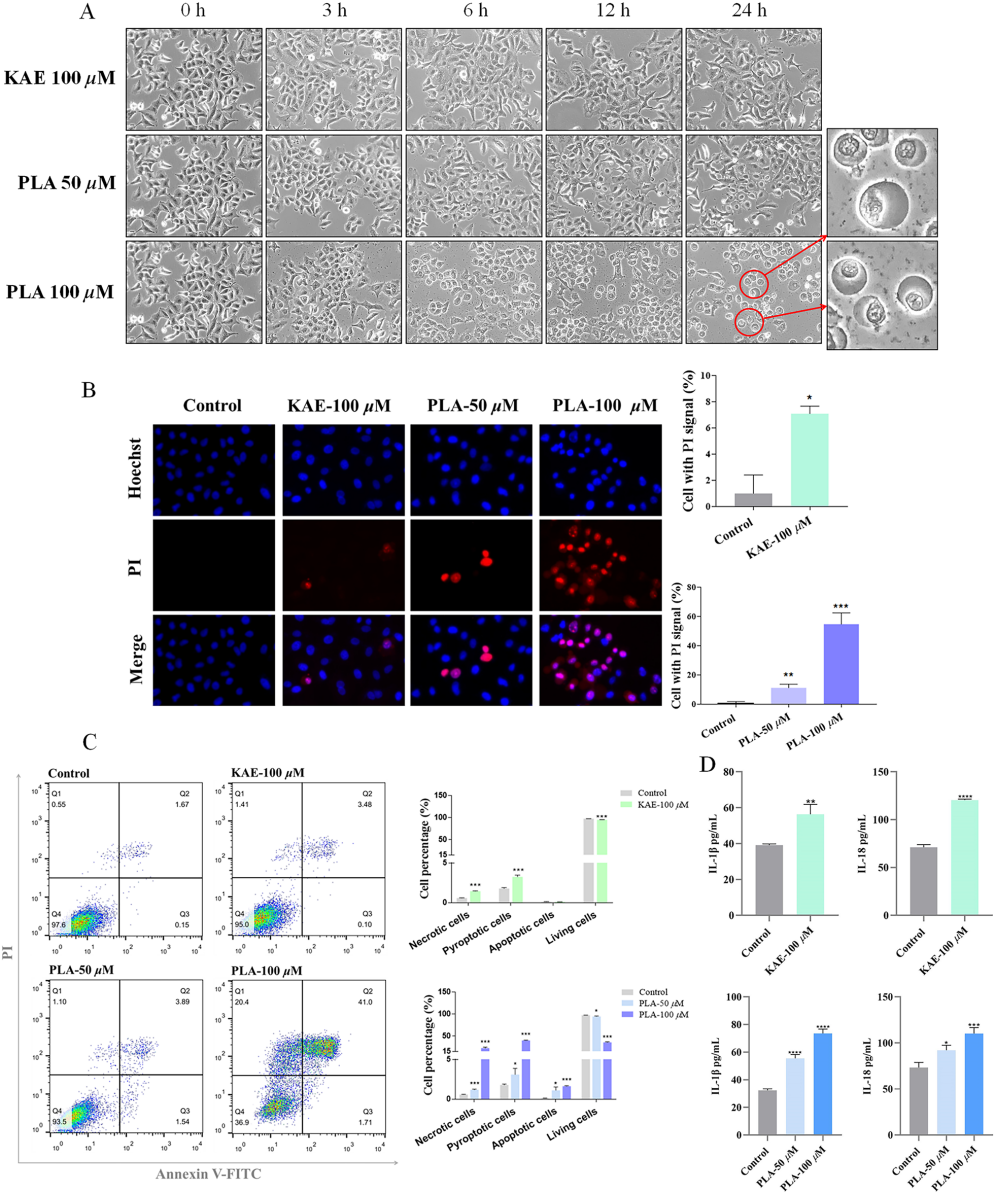
KAE- and PLA-mediated pyroptosis and upregulation of inflammatory factors in A549 cells. **A** Morphological effects of KAE and PLA on A549 cells: note cell swelling, formation of pore vesicles on the plasma membrane, and pyroptosis that was particularly obvious under 100 μM PLA. **B** and **C** Staining of A549 cells separately using Hoechst 33,342/PI (**B**) and Annexin V-FITC/PI (**C**) to examine the effects of KAE and PLA treatment on A549 pyroptosis. **D** ELISA of IL-1β and IL-18 expression levels in A549 cells. *P < 0.05, **P < 0.01, ***P < 0.001 vs. control group

Treatment of A549 cells with KAE or PLA led to a variety of responses over time, including cell swelling, the appearance of large membrane vesicles in monolayers, and the release of a large amount of cellular contents, indicating that the cells underwent pyroptosis (Fig. 4A). PLA at 100 μM led to the most pronounced changes in A549 cell morphology [39].

Hoechst 33342/PI combination staining (Fig. 4B) revealed that both KAE and PLA promoted A549 pyroptosis and increased the percentage of PI-positive cells, which was highest under 100 μM PLA. Annexin V-FITC/PI co-staining and analysis of pyroptosis using flow cytometry [40–42] (Fig. 4C) showed that treatment of A549 cells with KAE or PLA led to significant increases and decreases in the proportions of dead cells and viable cells, respectively, in the Q2 and Q3 zones of the scatterplot compared with the control group. Again, the most significant effect was observed 100 μM PLA. Furthermore, ELISAs demonstrated that KAE and PLA upregulated the expression levels of inflammatory factors IL-1β and IL-18 in the supernatants of A549 cell cultures (Fig. 4D).

### KAE and PLA exert anticancer activities in vitro through regulation of NLRP3 inflammasome pathway-associated proteins

**Fig. 5:**
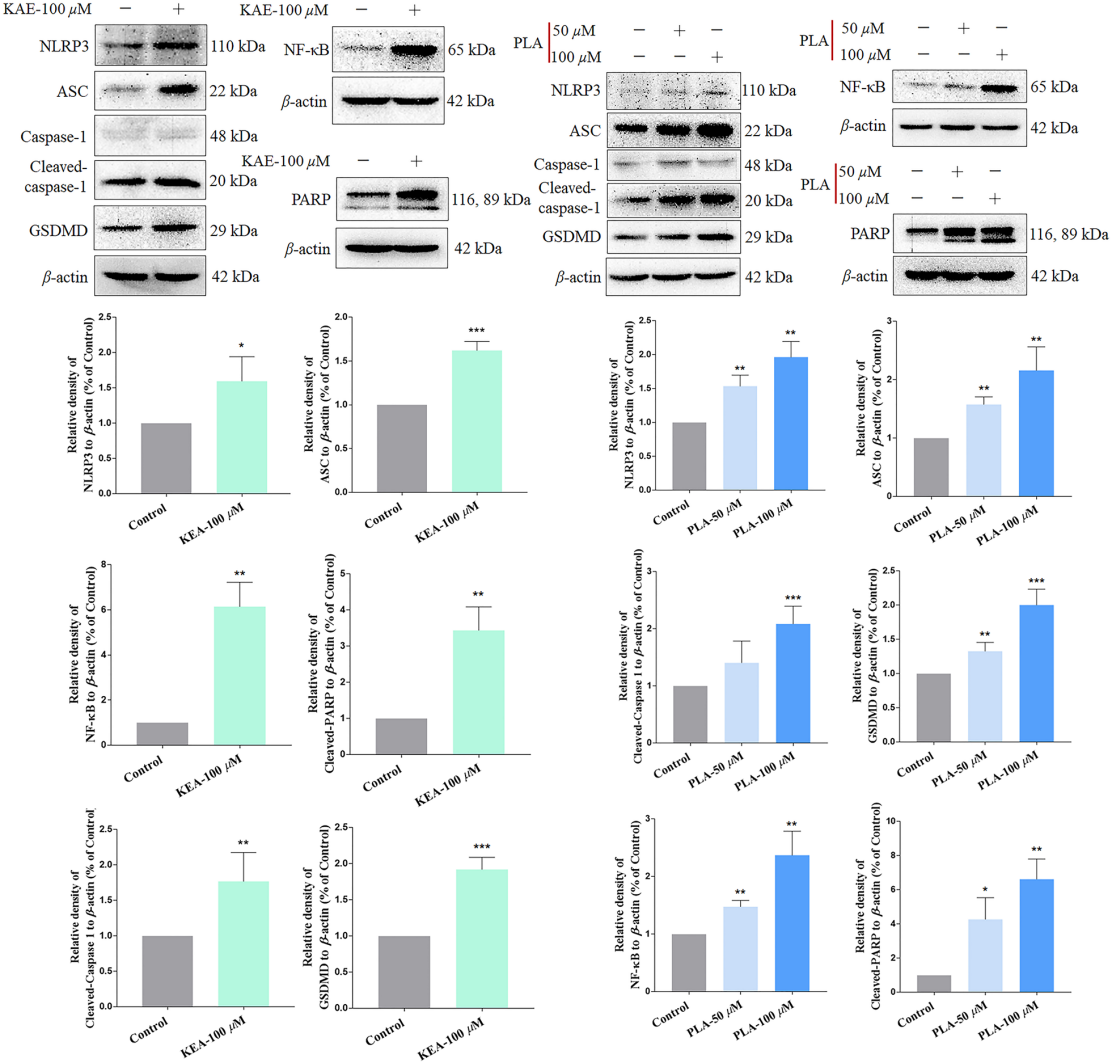
Effects of KAE and PLA on the expression of NLRP3 inflammasome pathway-associated proteins. Western blotting of NLRP3, ASC, caspase-1, GSDMD, NF-κB, and PARP in in A549 cells treated with KAE (100 μM) or PLA (50 μM and 100 μM). *P < 0.05, **P < 0.01, ***P < 0.001 vs. β-actin internal control

Western blotting was used to examine the effects of KAE and PLA on the expression of NLRP3 inflammasome pathway-associated proteins. As shown in Fig. 5, both factors upregulated the expression levels of NLRP3, NF-κB, ASC, PARP, cleaved-caspase-1, and GSDMD, a pathway-related protein. Furthermore, for PLA, the enhancement of expression of NLRP3 inflammasome pathway proteins was dose dependent (Fig. 5). Taken together, it appears that the KAE- and PLA-induced pyroptosis of A549 cells was induced in part through the activation and expression of the NLRP3 inflammasome, which had significant anticancer effects in A549 cells in vitro.

### KAE and PLA induce pyroptosis in A549 cells by triggering the activation of NLRP3 expression

**Fig. 6:**
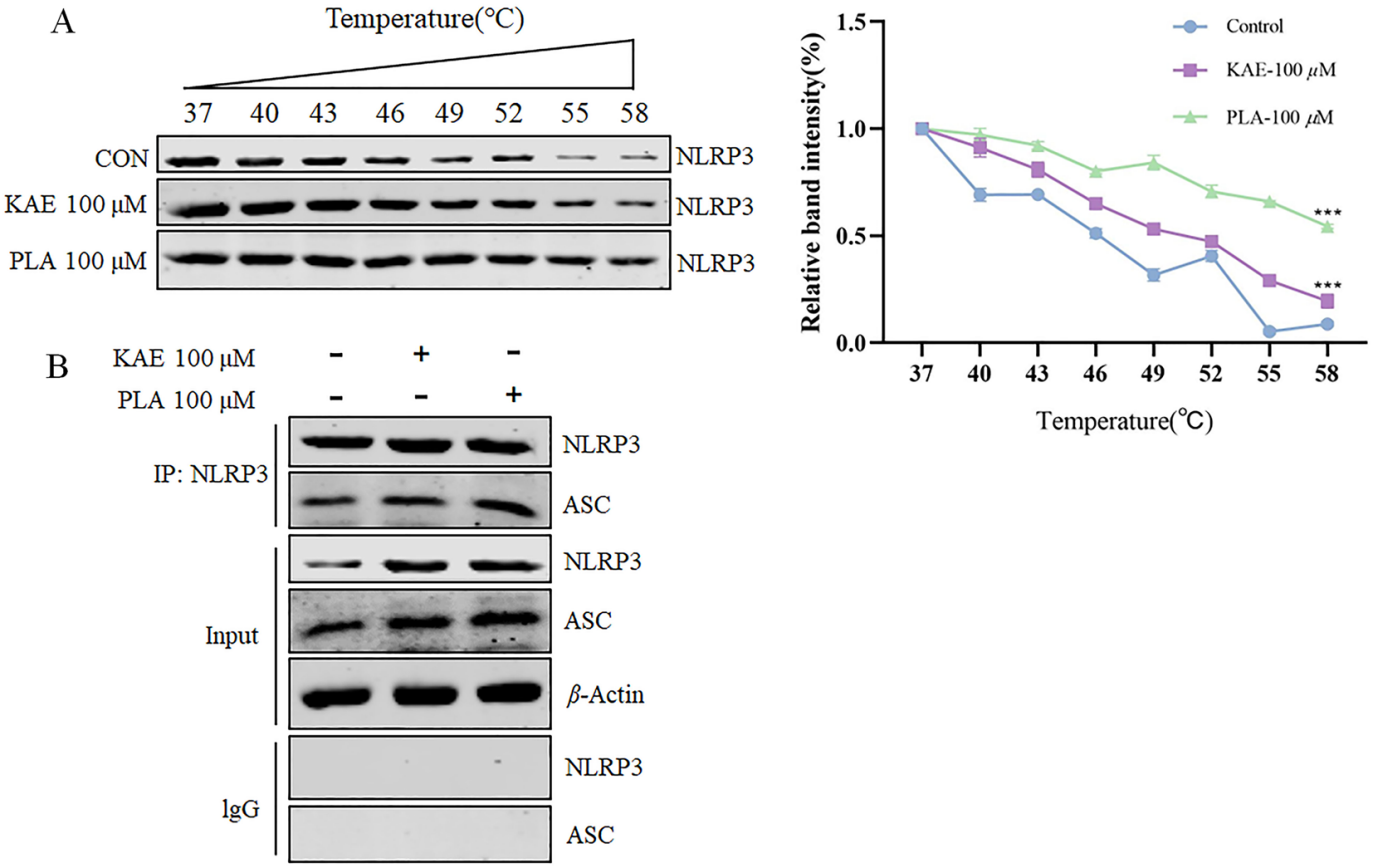
KAE and PLA exert their effects by targeting the NLRP3 protein and activating the NLRP3 inflammasome. **A** CETSA was employed to investigate the interaction between KAE, PLA, and NLRP3. **B** Immunoprecipitation studies further demonstrated that KAE and PLA not only up-regulated the expression of NLRP3 but also enhanced its binding to ASC. ***P < 0.001 vs. control group

The stability of a protein typically increases upon binding with a drug to the target protein, and the degradation of the target protein tends to decrease as the temperature rises. Exploring the underlying mechanism of KAE and PLA in promoting pyroptosis in A549 cells, we observed that the degradation of NLRP3 protein in the KAE and PLA group slowed down as the temperature increased, suggesting a potential association between drug-induced cellular pyroptosis and NLRP3 binding (Fig. 6A). To investigate this further, NLRP3 protein was immunoprecipitated using the immunoprecipitation technique to assess the expression of the ASC protein interacting with NLRP3. The results (Fig. 6B) revealed that both KAE and PLA upregulated NLRP3 expression and enhanced the binding of NLRP3 to ASC, facilitating the assembly of the NLRP3 inflammasome, consequently leading to the induction of pyroptosis.

## Discussion

In this study, two active components extracted from the flowers of *A. acutiloba*—KAE and PLA—were investigated for the first time, revealing potential roles for each in lung cancer treatment. An initial preliminary screening of these extracts showed that both compounds had inhibitory effects on the viability of A549 cells, leading us to screen for the effective concentrations of KAE and PLA. Inhibition of cancer cell proliferation in the early stage is the key to preventing the development of tumours. Therefore, we observed the changes in A549 cell viability under treatment with KAE or PLA for 24, 48, and 72 h, and also evaluated their inhibitory effects on cell proliferation via colony formation assay. Our findings showed that KAE and PLA significantly inhibited the proliferation of A549 cells in a concentration-dependent manner. Tumour metastasis is often the main reason for the failure of cancer treatment. To simulate the in vivo metastatic process of cancer cells, we evaluated the effects of KAE and PLA on A549 cells in vitro using the scratch assay. KAE and PLA significantly inhibited scratch healing, indicating that they may inhibit cell migration. Cells undergoing different types of death processes undergo distinct changes in growth status. Apoptotic cells crumple and become smaller, with the cell membrane remaining largely intact until it becomes an apoptotic body. By contrast, pyroptotic cells swell and become larger, with pore formation in the plasma membrane followed by rupture of the cell membrane and loss of integrity, which lead to the release of intracellular material and cause an inflammatory cascade. In this study, cell staining and flow cytometry were used to demonstrate that KAE and PLA induced pyroptosis in A549 cells.

**Fig. 7:**
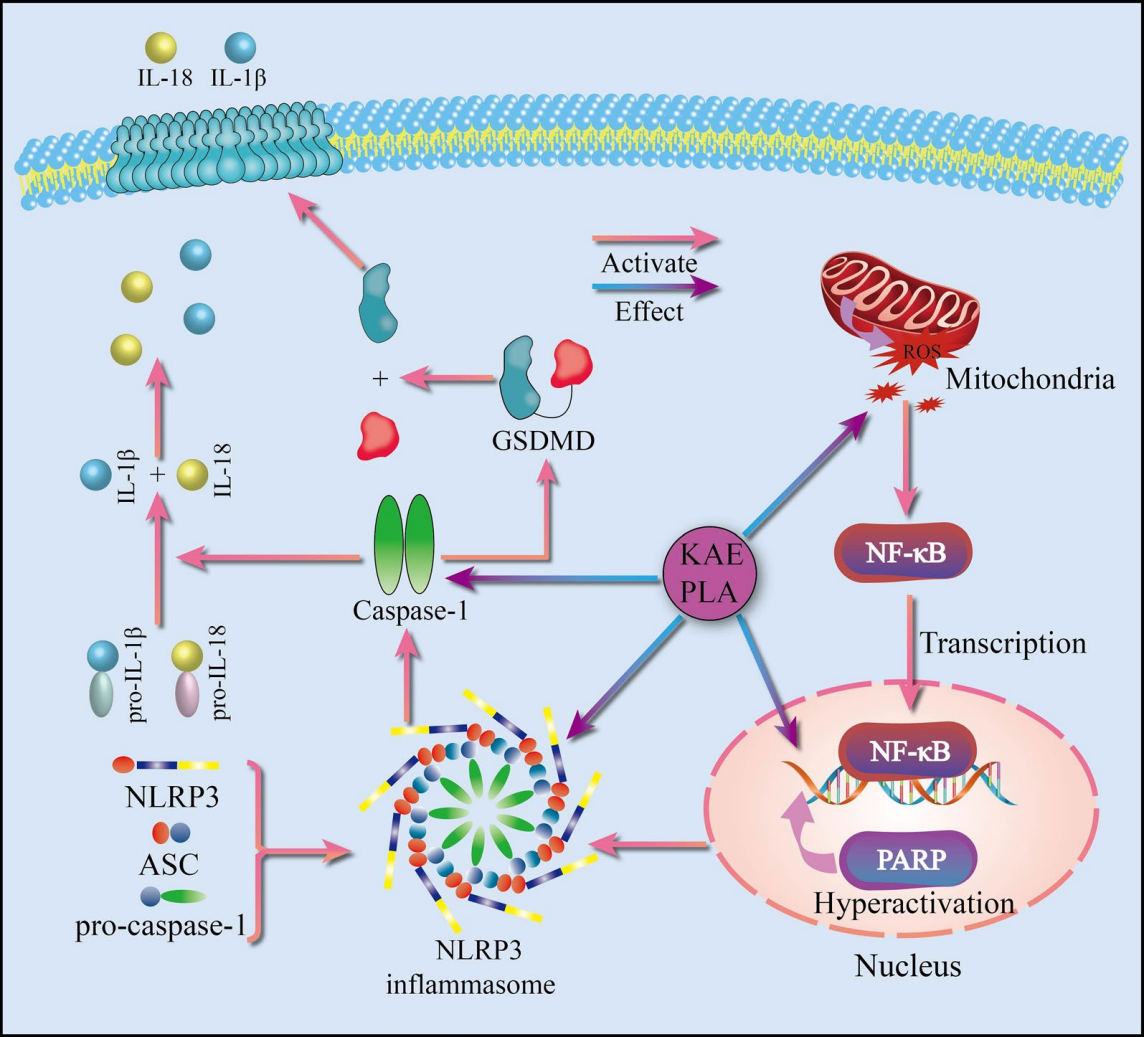
Mechanism of NLRP3 inflammasome activation and activities of associated proteins. KAE and PLA induce the NLRP3 inflammasome to assemble by increasing ROS levels, further upregulating NF-κB, NLRP3, and ASC protein expression, and thereby activating caspase-1 to shear its target protein GSDMD and induce pyroptosis

We also found that KAE and PLA caused oxidative stress in A549 cells, leading to the generation of ROS and activation of NF-κB [43], a transcription factor involved in a variety of important processes, including inflammation, immunity, and tumour development. NF-κB is an important regulator of the production of inflammatory factors and, upon activation, enters the nucleus to regulate transcription and translation, including the increased content of NLRP3 proteins [44]. NF-κB thereby mediates the NLRP3 inflammasome and triggers the activation of caspase-1, which then simultaneously reacts by shearing GSDMD, pro-IL-1β, and pro-IL-18 [45]. Shan et al. reported that, in lung cancer cells, sheared GSDMD forms pores in the cell membrane, leading to disruption of the ionic balance on both sides of the cell membrane, cell swelling and rupture, and ultimately pyroptosis [46]. In this study, we sought to verify their results by not only measuring the degree of release of cellular contents, but also by detecting proteins related to the NLRP3 inflammasome pathway. Our results showed that both KAE and PLA effectively increased the release of LDH from A549 cells, upregulating the expression levels of the pathway proteins as well as IL-1β and IL-18. A schematic diagram of this mechanism is provided in Fig. 7.

PARP is mainly found in the nucleus and acts as a sensor for cellular DNA damage, reflecting changes in cellular DNA. This protein is commonly used in apoptosis studies, but less so in pyroptosis studies [24, 25]. We sought to better understand the role of PARP in pyroptosis. Therefore, in our experiments, PARP was used as an indicator so as to observe the association between PARP and pyroptosis. We found that KAE and PLA upregulated PARP expression, implying that A549 cells died in response to treatment with each of the two compounds; however, the exact mechanisms by which KAE and PLA regulate this protein and lead to DNA damage require further investigation. Our findings provide a solid foundation for the development of KAE and PLA as therapeutic agents for lung cancer.

One potential limitation of our study of KAE with PLA against lung cancer is that it was limited to in vitro experiments at the cellular level. Further investigations are required to validate the anti-lung cancer effects of KAE and PLA in vivo.

## Conclusions

The chemical components of *A. acutiloba* flowers, namely KAE and PLA, demonstrated significant cytotoxic effects on lung cancer cells at administration concentrations of 50 μM and 100 μM. This led to the promotion of pyroptosis, thereby inhibiting the proliferation and migration of cancer cells and exhibiting potent anticancer activity in vitro. The mechanism underlying the anti-lung cancer effects of these two components was found to be related to the regulation of protein expression in the NLRP3 inflammasome pathway.

## Materials and methods

### Collection of *A. acutiloba* flowers and extraction and isolation of active compounds

Flowers collected from Longjing City, Yanbian Korean Autonomous Prefecture, Jilin Province, China, were identified as flowers of *A. acutiloba* by Professor Guanghai Shen, College of Pharmacy, Yanbian University. They are currently stored in the Specimen Library of the College of Pharmacy, Yanbian University (No. 20170327).

Dried *A. acutiloba* flowers (8.0 kg) were extracted using 95% ethanol (48.0 L × 3) and heating under reflux (2 h). The resulting ethanol extracts were decompressed and concentrated under reduced pressure to obtain a concrete (1.7 kg). The alcohol extract was mixed with water in equal volume and extracted three times to obtain a petroleum ether layer (215.6 g), an ethyl acetate layer (125.3 g), and an *n*-butanol layer (240.7 g), in this order. The ethyl acetate extract (95.0 g) of *A. acutiloba* flower was taken and subjected to gradient elution using normal-phase silica gel column chromatography with dichloromethane:methanol (100:0 ~ 0:100), from which 10 flow fractions (Fr1–Fr10) were obtained. From these, KAE (65.3 mg) and PLA (93.5 mg) were obtained using normal-phase silica gel column chromatography (dichloromethane:methanol) with Sephadex LH-20 (methanol) for the separation of Fr6 (5.4 g), and resolved by ^1^H-nuclear magnetic resonance (NMR) and ^13^C-NMR spectroscopy on an AV-300 (Bruker, Switzerland).

### Cell culture

The human NSCLC cell line A549 was obtained from the Kunming Cell Bank, Chinese Academy of Sciences. A549 cells were cultured using DMEM (containing 10% FBS and 1% PS (Gemini, USA)) medium (Hyclone, USA) and grown in an incubator at 37 °C with 5% CO_2_ in an incubator (Thermo, USA).

### Sample preparation

KAE and PLA were each solubilised using and diluted to prepare a series of concentration gradients. For treatment of cells, samples of each compound were diluted in medium such that the final concentration of DMSO in cell cultures was less than 0.1%.

Docetaxel, which was used as a positive control, was prepared by accurately weighing pharmaceutical-grade standard docetaxel (100666–201704, China Academy of Food and Drug Administration) and diluting to a 16 μM stock solution with DMSO [47]. For treatment of cells, the stock solution was diluted in medium to a final concentration of 16 nM.

### MTT assay for cell viability

A549 cells were grown to 80–90% confluence, then evenly distributed in 96-well plates, with an average of 1 × 10^4^ cells per well. After 24 h in 96-well plates, the cells were treated with docetaxel, a concentration gradient of KAE or PLA, with three replicate wells for each concentration. Cells were treated for 24, 48, or 72 h, then MTT reagent was added (2.5 mg/mL; Bio Froxx, Germany) for a 2 h incubation protected from light. The medium was aspirated and discarded, 100 μL DMSO was added to each well, and the plates were transferred to microplate reader (BioTek, USA) for measurement of optical density (OD) at 490 nm.

### Cell colony formation assay

A549 cells were grown to 80–90% confluence, and then evenly distributed in six-well plates at 3000 cells/well. After 24 h in six-well plates, the cells were treated with KAE or PLA for different time. The cultures were continued and regularly changed with fresh drug-free medium until the end of the culture on day 14 [48]. As the end of the experiment, the culture medium was discarded, and cells were washed and fixed for 60 min, then stained with crystalline violet (0.1% aqueous solution; China Shanghai Yuanye Biotechnology Co., Ltd., China) for 2 min. After washing the cells with depleted deuterium water, images were taken with a digital camera (Canon, China) under dry conditions at room temperature.

### Cell migration (scratch) assay

A549 cells 2.4 × 10^5^/well were cultured in six-well plates to ≥ 90% confluence. The monolayers were scratched, the medium was aspirated, the cells were washed with PBS, and culture was continued in KAE- or PLA-containing medium for all except the control group. Photographs of each group were taken at different time points for 24 h using an inverted integrated microscope (ECHO, USA).

### LDH release assay

A549 cells were evenly distributed in 24-well plates and treated with KAE or PLA 24 h later. At the end of the treatment, LDH assay working solution was added and incubated for 1 h. As a positive control, 100-μL aliquots of LDH release reagent was added to each well and mixed 1 h before the LDH assay (China Biyuntian Biotechnology Co., Ltd). Control wells with only culture solution were also set up as a negative control. At the end of the incubation, OD values were determined at 490 nm using microplate reader.

### Fluorescent dichlorodihydrofluorescein diacetate (DCFH-DA) assay of ROS

A549 cells were evenly distributed in 60-mm culture dishes containing 3 mL culture medium (5.4 × 10^5^ cells/dish). After 24 h, 3 mL fresh medium was added to the blank control group, and 3 mL KAE- or PLA-containing medium was added to the treatment groups [17] for 24 h. The treatment + medium was then aspirated, replaced with 1:1000 configured serum-free medium containing DCFH-DA, and incubated in the cell culture incubator for 20 min. Images were collected and analysed after washing the cells with serum-free medium.

### Detection of pyroptosis by flow cytometry

A549 cells were cultured in six-well plates at a density of 1.2 × 10^5^ cells/mL and treated with KAE or PLA 24 h later. The treated cells were then collected, washed, and re-suspended in 500 μL combined with a buffer. The cell samples were mixed with 5 μL annexin V-FITC dye (China Wan Class Biotechnology Co., Ltd., China) followed by 10 μL propidium iodide (PI) dye and incubated for 15 min at room temperature, protected from light, then analysed using a flow cytometer (Becton Dickinson Biosciences, USA).

### Hoechst 33342/PI fluorescence staining

Logarithmically grown A549 cells were evenly distributed in six-well plates and cultured for 24 followed by treatment with KAE or PLA for 24 h. To detect the effect of each treatment on pyroptosis, the medium was aspirated and discarded, and 5 μL staining buffer and Hoechst 33342/PI the two staining solutions were mixed successively, and incubated with the cells for 40 min, washed once with PBS, and observed under a fluorescent microscope (ECHO, USA).

### ELISA of inflammatory factors

Supernatants of KAE or PLA-treated A549 cell cultures were collected and centrifuged. The levels of IL-1β and IL-18 in the supernatants were detected using an assay kit (Jiangsu Meibiao Bio-technology Co., Ltd., China) and the OD values were measured at 450 nm using microplate reader.

### Cellular thermal shift assay (CETSA)

A549 cells were cultivated in 60 mm Petri dishes and processed in accordance with the previously described method [49]. Eight samples were collected from each group, with each sample measuring 100 µL. The samples were subjected to temperature incubation at 37, 40, 43, 46, 49, 52, 55, and 58 °C for 3 min, followed by two cycles of freezing and thawing in liquid nitrogen. Subsequently, the samples were centrifuged at 20,000 g at 4 °C for 10 min. The resulting supernatant was then mixed with sample buffer for Western blotting.

### Co-immunoprecipitation

A549 cells were harvested and gently lysed in RIPA buffer for 15 min. The lysed samples were quantified and then aliquoted using a BCA kit (Solarbio, China). Target antibodies or IgG were introduced to the individual samples, followed by an overnight incubation at 4 °C. Subsequently, 15 μL of magnetic beads were incorporated into the samples and incubated at room temperature for 1 h. After a PBS wash, Western blotting was conducted by adding the sample buffer.

### Western blotting

A549 cells were cultured on plates for 24 followed by treatment with KAE or PLA for 24 h, then used to prepare protein samples. BCA kit was used to quantify the protein concentrations. An appropriate amount of each protein sample was selected for polyacrylamide gel electrophoresis, and then transferred to polyvinylidene fluoride membranes, which were blocked for 1 h and incubated with primary antibody overnight at 4 °C with slow shaking. The membranes were then washed with TBST and immersed in secondary antibody working solution for 1.5 h. Antibody dilution ratios are provided in the Additional file 1: Table 1. Spots were detected using a gel imager (ProteinSimple, USA) and analysed in grey scale using ImageJ 8.0 software.

### Statistical analysis

Data were statistically analysed and graphed using GraphPad Prism 7.0. At least three sets of data were collected from each experiment and are expressed as means ± standard deviation. The significance levels of between-group differences were analysed using one-way analysis of variance or paired *t*-tests.

### Supplementary Information

**Additional file 1: Table 1**. Methods of primary antibody and secondary antibody dilution.

## Data Availability

All the data generated or analyzed during this study are included in this article. Further inquiries can be directed to the corresponding author.
